# Pain and Laboratory Animals: Publication Practices for Better Data Reproducibility and Better Animal Welfare

**DOI:** 10.1371/journal.pone.0155001

**Published:** 2016-05-12

**Authors:** Larry Carbone, Jamie Austin

**Affiliations:** Laboratory Animal Resource Center, University of California San Francisco, 513 Parnassus, San Francisco, California 94143-0564, United States of America; Central South University, CHINA

## Abstract

Scientists who perform major survival surgery on laboratory animals face a dual welfare and methodological challenge: how to choose surgical anesthetics and post-operative analgesics that will best control animal suffering, knowing that both pain and the drugs that manage pain can all affect research outcomes. Scientists who publish full descriptions of animal procedures allow critical and systematic reviews of data, demonstrate their adherence to animal welfare norms, and guide other scientists on how to conduct their own studies in the field. We investigated what information on animal pain management a reasonably diligent scientist might find in planning for a successful experiment. To explore how scientists in a range of fields describe their management of this ethical and methodological concern, we scored 400 scientific articles that included major animal survival surgeries as part of their experimental methods, for the completeness of information on anesthesia and analgesia. The 400 articles (250 accepted for publication pre-2011, and 150 in 2014–15, along with 174 articles they reference) included thoracotomies, craniotomies, gonadectomies, organ transplants, peripheral nerve injuries, spinal laminectomies and orthopedic procedures in dogs, primates, swine, mice, rats and other rodents. We scored articles for Publication Completeness (PC), which was any mention of use of anesthetics or analgesics; Analgesia Use (AU) which was any use of post-surgical analgesics, and Analgesia Completeness (a composite score comprising intra-operative analgesia, extended post-surgical analgesia, and use of multimodal analgesia). 338 of 400 articles were PC. 98 of these 338 were AU, with some mention of analgesia, while 240 of 338 mentioned anesthesia only but not post-surgical analgesia. Journals’ caliber, as measured by their 2013 Impact Factor, had no effect on PC or AU. We found no effect of whether a journal instructs authors to consult the ARRIVE publishing guidelines published in 2010 on PC or AC for the 150 mouse and rat articles in our 2014–15 dataset. None of the 302 articles that were silent about analgesic use included an explicit statement that analgesics were withheld, or a discussion of how pain management or untreated pain might affect results. We conclude that current scientific literature cannot be trusted to present full detail on use of animal anesthetics and analgesics. We report that publication guidelines focus more on other potential sources of bias in experimental results, under-appreciate the potential for pain and pain drugs to skew data, and thus mostly treat pain management as solely an animal welfare concern, in the jurisdiction of animal care and use committees. At the same time, animal welfare regulations do not include guidance on publishing animal data, even though publication is an integral part of the cycle of research and can affect the welfare of animals in studies building on published work, leaving it to journals and authors to voluntarily decide what details of animal use to publish. We suggest that journals, scientists and animal welfare regulators should revise current guidelines and regulations, on treatment of pain and on transparent reporting of treatment of pain, to improve this dual welfare and data-quality deficiency.

## Introduction

Scientists conduct animal surgeries daily in laboratories around the world. They plan in advance for managing the pain these surgeries are likely to cause. What information on animal pain management will a reasonably diligent scientist find in planning surgeries for a successful experiment? Most often, while a surgery may be necessary to the experiment, the pain it causes is not. [1] Scientists must actively choose the surgical anesthetics and post-surgical analgesics they administer. Sometimes they choose to leave the animals’ pain untreated, concerned that eliminating the pain of animal surgeries requires the use of potent anesthetic and analgesic drugs whose effect on the animals’ biology goes beyond simply deadening the pain. They may determine that the need to avoid the data artifacts such potent drugs can cause may outweigh the pain that animals may experience. Given the welfare concerns of under-treated animal surgical pain, and scientists’ concerns to minimize the unwanted experimental artifacts of potent drugs and of untreated pain itself, scientists should explicitly describe pain management every time they publish work that involved animal surgery. In this project, we analyze how scientists do, and mostly, do not, describe and discuss choices in animal pain management.

Pain management for animals in laboratories is an ethical imperative. It is also a methodological challenge for scientists, as surgical anesthetics, potent post-surgical pain medications, and untreated pain itself, all affect the animals’ biology in profound ways. Pain and pain-relieving drugs both can skew experimental data, introducing unwanted variability or sources of confound.[2, 3] These entwined concerns—that both pain and pain drugs can affect both experimental outcomes and the animals’ welfare—should lead scientists writing their experimental research papers to carefully describe pain management practices in their research methods.

If scientists who conduct major surgeries on animals all described their choice of surgical anesthetics and post-surgical analgesics, along with any decisions not to treat post-surgical pain, science and animals would both benefit. Truly informative systematic reviews and meta-analyses of the effects of pain, anesthesia and analgesia on research outcomes would be possible if readers had access to this information, and these reviews could help scientists standardize reproducible methods. Scientists forced to explain their decision to leave surgical pain untreated might be more inclined to try their experiments with analgesics included. If scientists saw their peers clearly describing animal pain management, they could be confident that pain treatments are possible in their own experiments and can be consistent with quality data finding its way into high caliber journals.

Biomedical scientists are both the authors and the audience of the literature in their field. As authors, they edit their articles to include only those methodological details they believe to be crucial to interpreting the solidity of their findings. As readers, they assess each other’s findings and may build on each other’s methods. They learn the standard of animal care in their field from these published papers, and look to this literature when writing and justifying protocols for their Institutional Animal Care and Use Committee (IACUC) or animal ethics committee. The United States Department of Agriculture (USDA), in enforcing the Animal Welfare Act (AWA), considers a search of the literature as “the most effective and efficient method for demonstrating compliance with the requirement to consider alternatives to painful/distressful procedures.”[4] A database search is no stronger than the literature in the field it searches. Does the literature in their field give them enough detail on anesthesia, analgesia and pain to use it for these purposes? Or is that information edited out as methodologically insignificant? If a paper’s description of an animal surgery does not include anesthesia and analgesia details, a reader may conclude, rightly or wrongly, that pain medications are not necessary, or even that they could invalidate the experiment or its data.

Several authors and groups have reviewed how animal researchers describe their animal experiments. Some focus on animal welfare refinements and pain management, with little overt discussion of best practices for reliable, reproducible data.[1, 5–13] Others have critiqued the animal research literature for concerns with transparency, data-quality and reproducibility, highlighting sources of bias that have also plagued human subjects research, such as randomization, robust statistics, and blinding of data-collectors to group allocations.[9, 14–29] Recent guidelines for publishing animal studies, most notably from the UK National Centre for the 3Rs and the United States National Academies of Science, suggest standards for fuller and more consistent reporting of animal experimental methods in scientific articles, but with very little attention to animal pain management as a methodological practice of concern.

In this project, we synthesize these two strands of analysis, focusing on animal pain management as both an issue of quality, reproducible data and as a serious animal welfare concern. If scientists under-report their use of analgesics, or under-treat animal pain, the impression of analgesic non-use can perpetuate further analgesic non-use among scientists seeking to replicate others’ studies or to borrow their methods. Under-reporting the details of pain management may erroneously edit out important methodological information, as both pain medications and untreated pain both have whole-body metabolic, immunologic and other effects that can affect animal research data.[3, 30–32] Readers need to know whether pain was treated after surgeries, and even which particular anesthetics and analgesics were used, if they are to critically evaluate, reproduce or build upon published work.

**Fig 1:**
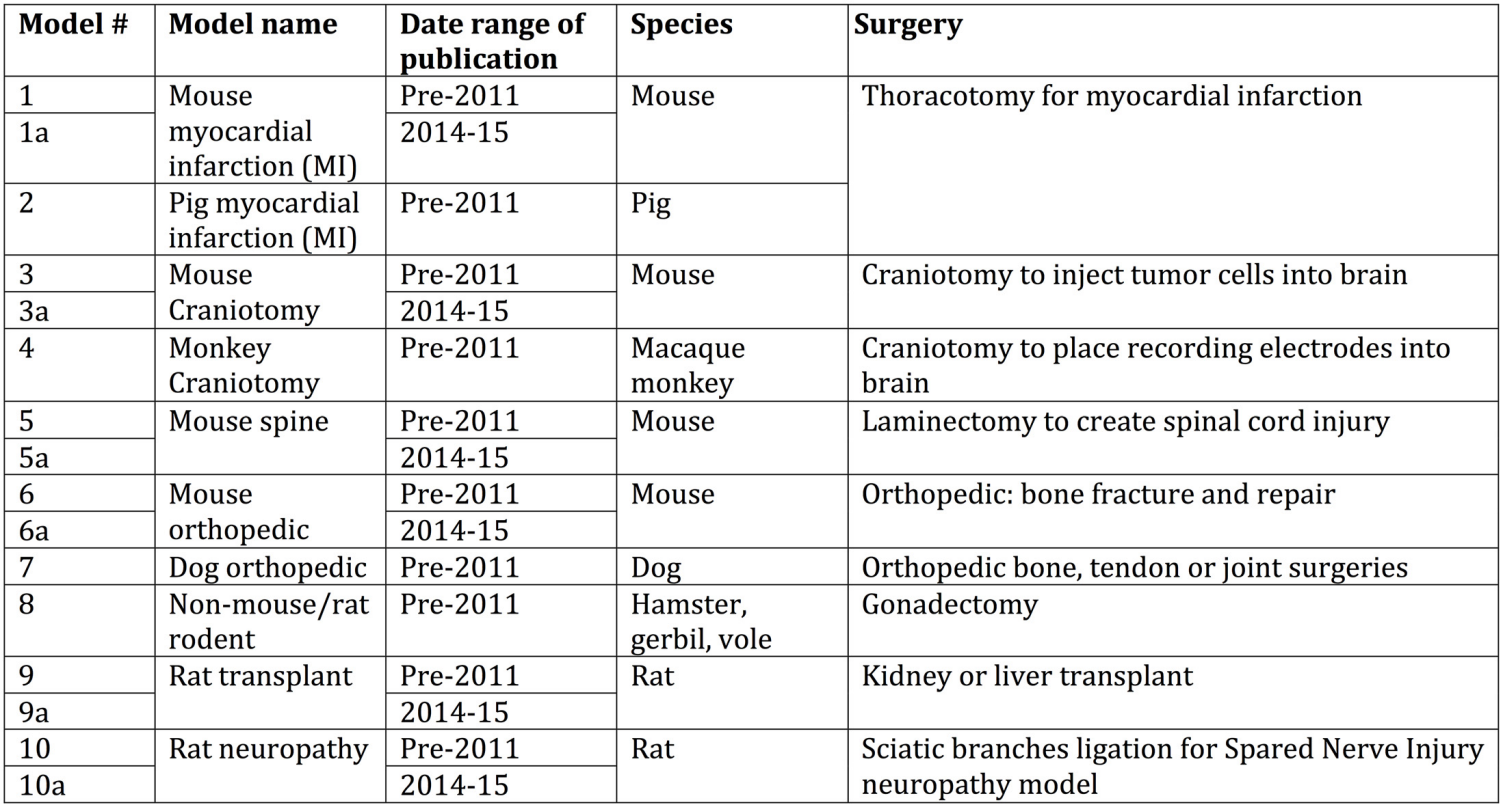
Models Scored for Publication Completeness (PC), Analgesic Use (AU) and Analgesic Completeness (AC). 25 articles per model.

In this project, we evaluated the completeness of reporting on animal pain management in 400 articles a range of animal research fields. To do this, we scored papers that used 10 different surgical models for the completeness of their descriptions of anesthesia, analgesia and for any justifications for withholding pain treatments (Fig 1). The ten surgeries were all performed as the means to an end, that is, the scientist had to perform a surgery to alter physiology (e.g., gonadectomy), create an injury (e.g., spinal, limb, heart or peripheral nerve injuries), or access deep tissue (brain cells) in order to go on with the experiment. We examined liver and kidney transplants where the biology of graft rejection, not a new surgical or anesthetic methodology, was the focus of the research. Surgery, and the anesthesia it requires, were necessary background procedures to model human health conditions that are themselves not caused by surgery; surgery and pain management were not the independent variables under examination.

The surgeries in these 10 models were all sufficiently involved and invasive that technical needs alone required general anesthesia, even if only to keep the animal still. If the scientist did not mention anesthesia in our 10 models, we are confident that that was an editorial decision, not a failure to anesthetize the animals. Post-surgical analgesia and pain management, on the other hand, may affect research outcomes, but they are not technical requirements. In most cases, the animals will survive their treated or untreated post-operative pain, and the scientist will have the lesioned organ, the recording electrodes, the tumors in the brain needed for the experiment. Whereas anesthetized surgeries are the means to the research ends (creating neurological or heart lesions, etc.), analgesic drugs are the means to a different end: the ethical and regulatory obligation to minimize animal pain. Because anesthesia is a stronger technical requirement than analgesia, the first two questions scientists consider in planning for anesthesia and analgesia are 1) *which* anesthetic to use, and 2) *whether* to use analgesics. “Which analgesic?” is only a question once the “whether to use” question has been resolved.

This project weaves together concerns for quality science and for animal welfare. We believe that if the literature a scientist finds in planning her experiments is confusing on questions of animal pain management, the quality of her data, her success at replicating others’ findings, and the animals’ welfare will all suffer. We took a consumer’s viewpoint in reviewing 400 published articles using 10 different animal models: what information will a reasonably diligent scientist find in planning for a successful experiment and an approvable IACUC protocol?

## Materials and Methods

We designed our literature review to simulate what research scientists would do, whether in looking to replicate and use a particular experimental model in their work, or in a good faith effort to search for humane alternatives, including refined pain management, to painful procedures in their laboratory animals. In contrast to a structured literature review, we assumed a scientist would focus on current literature, rather than reading all the possibly relevant articles in the field, and would also follow up on cited references, rather than requiring all information to be in stand-on-their-own primary articles.

We identified 10 models, or experimental procedures, all of which entail surgery on animals, and all of which are too technically challenging to perform on an unanesthetized subject. (See Fig 1 for list of models) We used Pubmed Central as our resource. In our first review, to exclude the influence of 2011 ARRIVE and National Academies guidelines on publication of animal studies, we only reviewed articles accepted for publication before December 31, 2010.[16, 18] Where the date of acceptance for publication was not clear, we excluded articles first published electronically in 2011 or copyrighted in 2011. We scored articles for the completeness of their reporting on anesthesia and analgesia. We scored the 25 papers in each model that met our inclusion criteria for date of publication, availability, language, and availability of cited literature, in order of recent addition to the PubMed database.

To examine whether the 2010–11 guidelines affected subsequent publication practices, we scored an additional 25 articles, published in 2014–15, in the six of the 10 models that use laboratory rats or mice, bringing the total of formally scored articles to 400.

In addition to date of publication, our inclusion criteria included:

- Full text available in English through Galen II, UCSF’s on-line library system
- The article must report experimental data using the model as a means to performing experiments (not a review article, and not a description of how to anesthetize animals for the surgery)
- If authors left a gap or gaps in their paper in their description of anesthesia and/or analgesia, but stated they used methods described elsewhere, the cited article(s) must also be available full text and in English; these cited articles could be experimental reports themselves, or published “how to” methods papers. 163 articles required looking at cited articles. In some cases, we traced articles citing other articles back through 4 or more layers, until we found articles that either: 1) provided anesthesia and/or analgesia information that closed any information gap; 2) did not close gaps but also did not cite any others’ cited methods; or 3) that failed to meet our language and availability criteria. We did this until we scored the paper as having neither a Publication Gap or an Analgesia Gap. If the cited article did not actually use the same surgical procedure or the same species of animal, we did not use information from that article. If two or more cited articles contained conflicting information (e.g., two different anesthetic regimens) we scored the publication as “complete” but with unspecified anesthesia or analgesia. If following citations led us to articles that did not meet our inclusion criteria, we excluded the primary scored article from our consideration.

### Scoring

A single individual (LC), a laboratory animal veterinarian, scored all 400 articles.

First, we scored whether authors mentioned anesthetizing their animals at all, directly, or in their cited references. Papers that failed to report necessary anesthesia were scored as having a Publication Gap. Papers that reported the necessary anesthesia (n = 336) or described post-surgical analgesia without mentioning anesthesia (n = 2) were Publication Complete (PC = 1; n = 338).

We scored papers (including the references they cite) that stated use of anesthetics as well as post-surgical analgesics either as being Analgesia Users (AU = 1). Of the 338 Publication Complete articles, 240 (71%) had were Analgesia Non-users; these were further analyzed for whether they explicitly stated and explained that they did not use analgesics, or were silent on the question.

98 articles were Analgesia Users that stated use of post-operative analgesics. Of these, 6 did not specify their choice of analgesic, while 92 did. We then scored the 92 articles that named their choice of analgesics for how well they matched our scale of Analgesia Completeness (AC) based on American College of Laboratory Animal Medicine and National Academies of Sciences criteria for ideal pain management.[3, 33]

**Fig 2:**
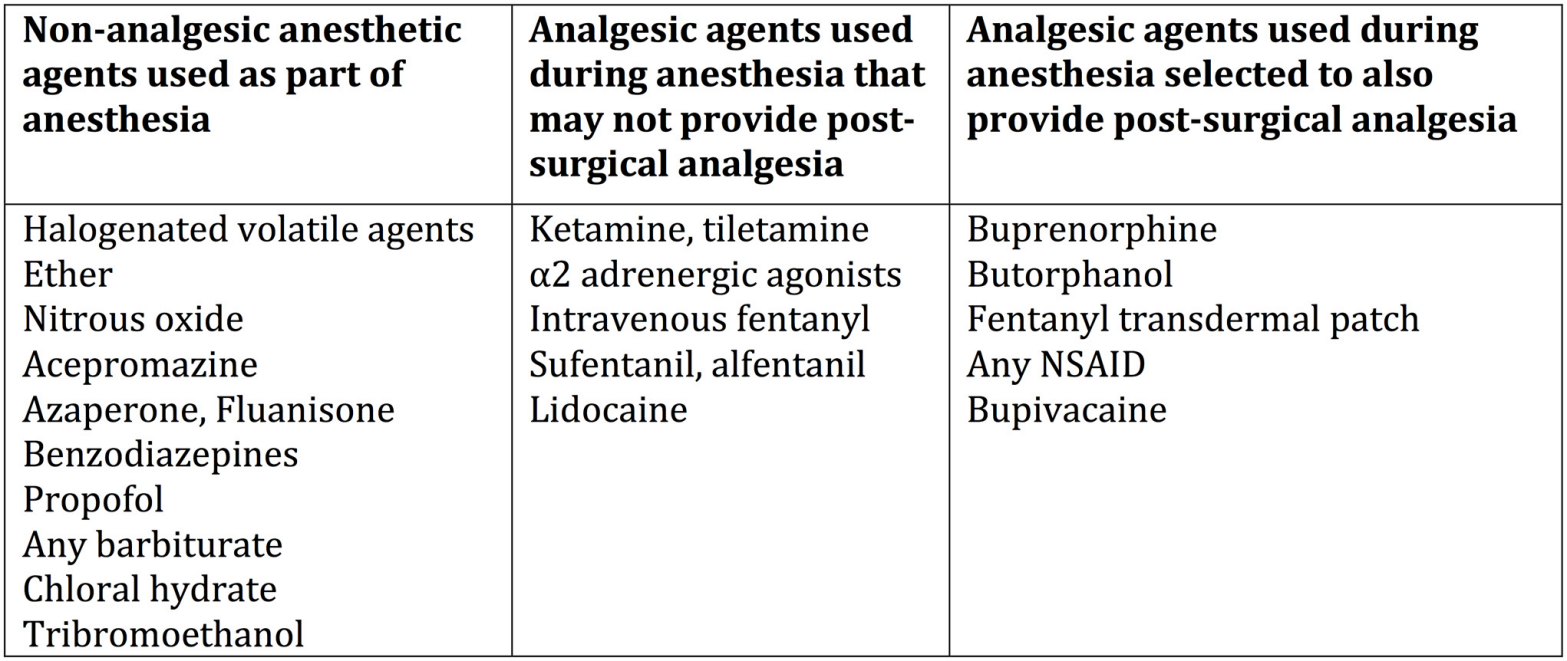
Analgesic Properties of Agents Used During Surgical Anesthesia.

We scored articles and awarded one point for each of these criteria, for a maximum of 4 points on our Analgesic Completeness scale:

- Any use of post-surgical analgesia
- Use of an analgesic agent as part of surgical anesthesia (balanced anesthesia in which a component of the surgical anesthesia includes an agent with analgesic efficacy–Fig 2)
- Multimodal use of two or more classes of analgesic agents for post-surgical analgesia
- Analgesics re-administered at least once post-surgically (if article was silent on this, it was scored as using a single dose of post-operative analgesic)

We did not formally score the particular choices of anesthetic and analgesic drugs.

There was no effect of date of publication (pre-2011 vs. 2014–15) on either Publication Completeness or Analgesia Use for the 6 rat/mouse models. Therefore we included the 150 2014–15 papers in the dataset of 400 articles.

### Journals

Based on information available in mid-2015, we reviewed whether the journals containing our articles explicitly state in their instructions for authors adoption of the use of the ARRIVE Guidelines and/or are listed by the National Centre for the 3Rs (publishers of the ARRIVE Guidelines) as adopters. We also reviewed the 2013 Impact Factor (when available) for each journal (recognizing that the Impact Factor could have changed slightly from 2010 to 2015). We only scored the journal in which the article we were scoring was published, including in cases where we had to go to cited papers for the anesthesia and analgesia details that we credited to the primary, formally scored paper.

Journal Impact Factors change slowly enough that we chose to score all 400 articles for whether the 2013 Impact Factor had an effect on PC, AU or AC. We excluded the 4 articles published in 4 journals (*Medical Science Monitor Basic Research*, *Physiological Reports*, *Frontiers in Neuroscience*, and *Stem Cell Reports*) that were too new to have Impact Factors available.

We further scored the 150 2014–15 articles for whether reference to ARRIVE or NAS guidelines had an effect on PC or AU. For this, we examined the journals’ instructions for authors for reference to ARRIVE, NAS or EQUATOR guidelines (which explicitly endorse ARRIVE guidelines).[34] 93 journals published these 150 articles (1 to 13 articles per journal; mean = 1.6; median = 1) 45 articles were published in 25 journals with author instructions to consult these publication guidelines.

**Fig 3:**
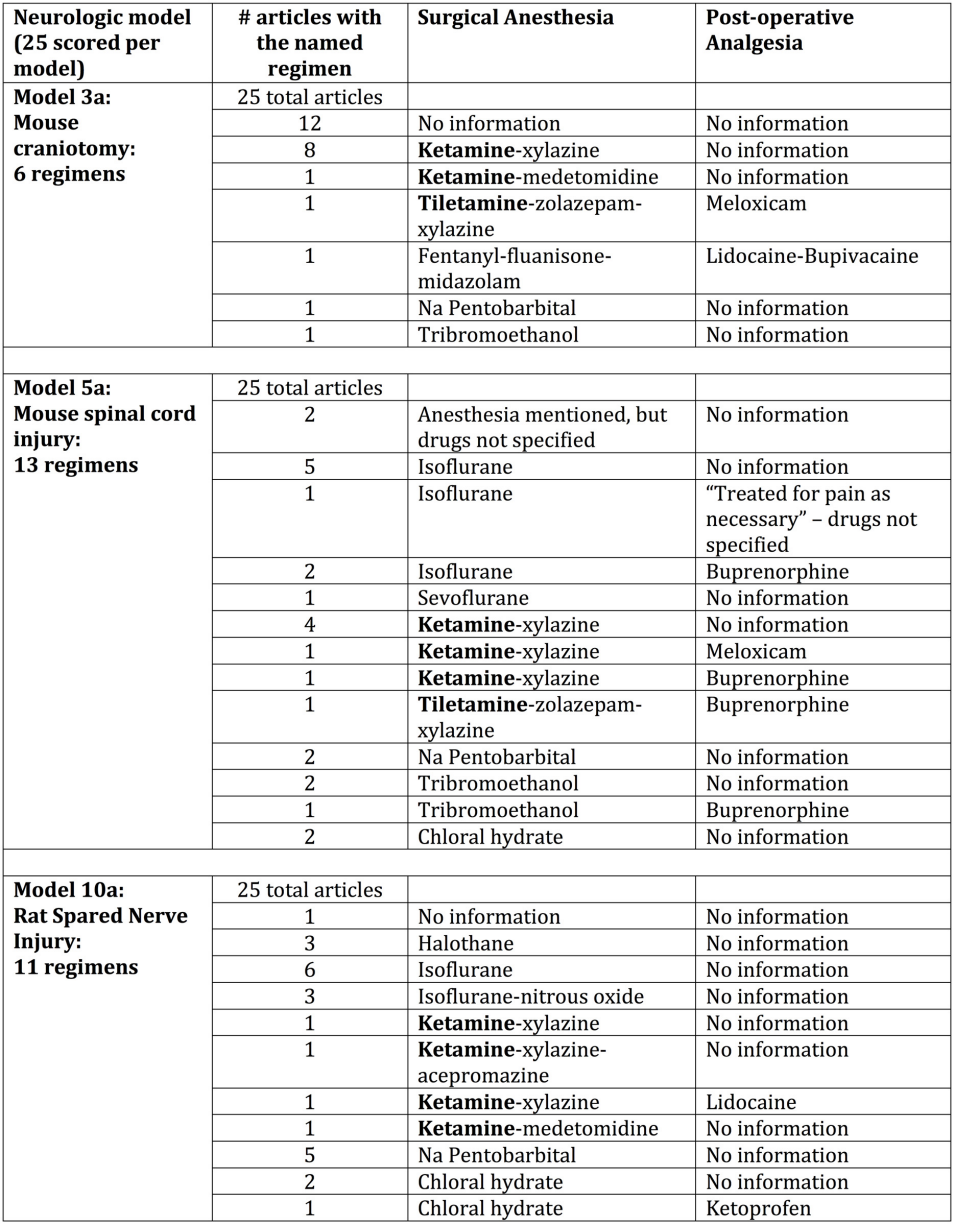
Anesthetic-Analgesic Regimens in Neurological Model Articles in the 2014–15 Publication Range. Use of ketamine and tiletamine are bolded; these agents, which are recommended against for neurological models by the CAMARADES guidelines.

The CAMARADES checklist for publication of neuroscience models includes “avoidance of anesthesia with intrinsic neuroprotective properties” as one of its 10 items.[21, 35] It lists ketamine as its sole example of a neuroprotective anesthetic, but does not provide more extensive review of neuroprotective anesthetics or any discussion of analgesics’ neuroprotective qualities. We listed the anesthetic-analgesic regimens used in the 75 2014–15 rodent neurological articles we scored (models 3a, 5a and 10a). We noted those that used ketamine or its near-relative tiletamine, but were unable to further score compliance with CAMARADES as its list of neuroprotective agents is incomplete. In Fig 3, we scored non-mention of post-surgical analgesic as one regimen, “no information,” though it might combine analgesics non-use articles in with analgesics-used-but-not-described articles. For simplicity of review, we did not score single-dose and repeated-dose of analgesics as separate regimens.

### Statistics

RStudio Version 0.99.486 (2015) was used for all analyses.[36]

## Results

We scored 250 articles accepted for publication before 2011 in 10 models, and 150 2014–15 publications in 6 of those models. (Fig 1) The complete spreadsheet of all 400 articles is available in Supplemental Materials.

We scored these articles for:

- The article (and/or qualifying articles it cites) includes any mention of anesthesia or analgesia for the particular surgical procedure (Publication Complete = PC): 338 articles
- Of the 338 PC articles, the article includes any mention of post-surgical analgesia (Analgesic Use = AU): (98 articles)
- Analgesic completeness (scale of 1 to 4) for 92 articles that name the specific analgesics used (Analgesia Completeness = AC)

The 6 mouse-rat models were analyzed in both time periods.

### Publication with any mention of surgical anesthesia or post-surgical analgesia (Publication Completeness; PC = 1)

**Fig 4:**
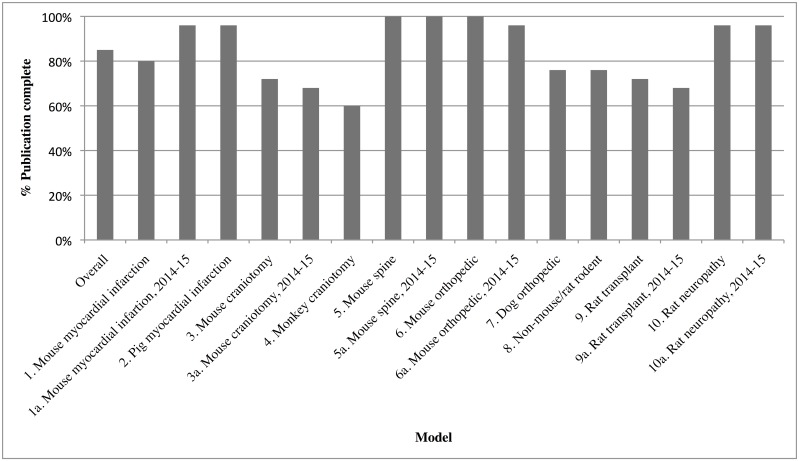
Publication Completeness per Model. The percentage of 25 scored articles in each model that mention use of surgical anesthesia and/or post-surgical analgesia.

Publication Completeness scores for the 10 models in both time periods are presented in Fig 4. Two articles stated use of analgesia but were silent on the fact that the animals were also anesthetized. For analysis, we grouped these two articles as PC with those that mentioned both anesthesia and analgesia.

Overall, PC for the 400 articles was 338/400 = 85%, with no difference by publication date. Mouse or rat models (300): 261/300 = 87%. Non mouse-rat rodents (model 8): 19/25 = 76%. For non-rodents (models 2, 4and 7), PC was 58/75 (77%).

### Post-surgical Analgesia Use (AU = 1)

338 of 400 articles reported some use of anesthesia or analgesia, and were evaluated for their use of post-operative analgesia (AU). We scored articles that described their surgical anesthesia without mentioning post-operative analgesia as having an analgesia gap (AU = 0), including those whose surgical anesthesia included agents (such as fentanyl, ketamine and lidocaine) that have short-acting analgesic qualities; an article showed Analgesia Use (AU = 1) only if it included analgesics expected to have extended analgesic affects after the animals awakened from anesthesia.

**Fig 5:**
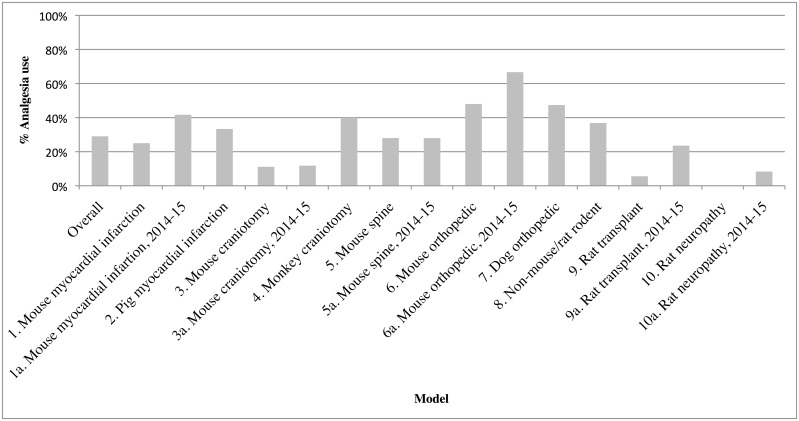
Analgesia Use per Model. The percentage of Publication-Complete articles in each model that mention use of post-surgical analgesia.

Overall, AU was 98/338 = 29%. (Fig 5) For mouse or rat models, analgesia use was 68/261 = 26%. Non mouse-rat rodents (model 8): 7/19 = 37%. For non-rodents(models 2, 4and 7), AU was: 23/58 = 40%.

### Analgesic Completeness

**Fig 6:**
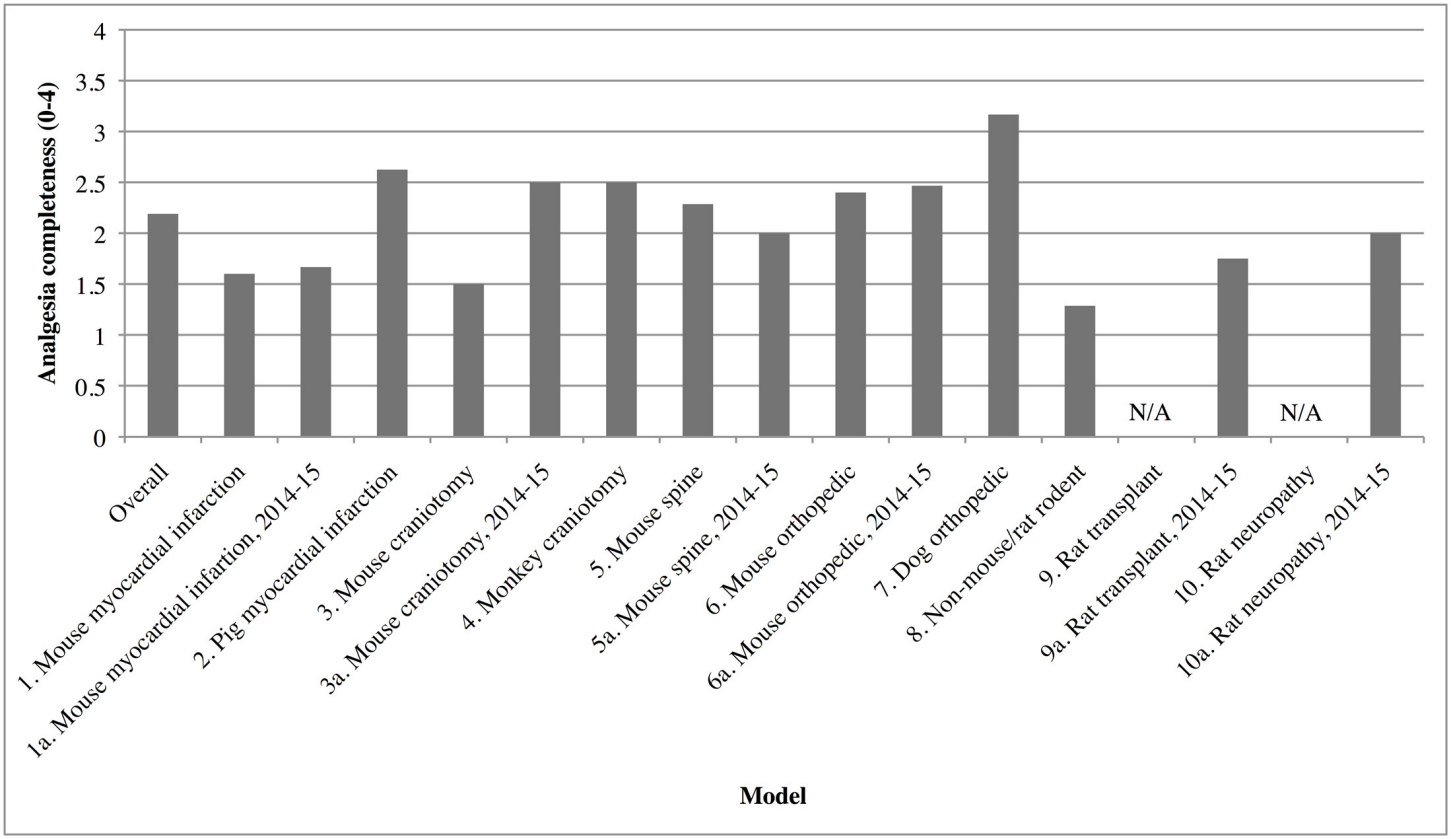
Mean Analgesia Completeness per Model, on a Scale of 1 to 4. Articles that are scored as Analgesia Users (AU) and specify their post-surgical analgesic regimen receive one point each for: any mention of post-surgical analgesia; multimodal use of 2 or more classes of post-surgical analgesics; use of an analgesia agent during surgical anesthesia; and analgesic re-administration at least once post-surgically. No articles in Models 9 or 10 included sufficient detail to allow for scoring of AC.

92 of 98 AU articles named their analgesics; six made a generic statement of analgesic use, without specifying the analgesic’s name, dose or frequency. We scored those 92 articles for Analgesic Completeness (AC) on a scale of 1–4. (Fig 6) Four articles received a score of 4, 27 articles received a score of 3, 40 articles received a score of 2 and 17 articles received a score of 1. No articles in Models 9 or 10 included sufficient detail to allow for scoring of AC.

Multimodal analgesia was uncommon, and the main reason for receiving a score below 4 was the low use of multimodal analgesia: only 7 of the 92 papers mentioned use of 2 classes of post-operative analgesic, and 1 used 3. Of the 8 articles with multimodal analgesia, 6 combined an opioid with an NSAID or acetaminophen, 1 used an opioid and a short-acting local block (lidocaine) and 1 dog orthopedics article combined tramadol with NSAID and an unspecified “IV analgesic,” which would likely be an opioid.

### Non-use or non-mention of post-surgical analgesia

240 PC articles that mentioned anesthesia did not mention analgesia. None of these explicitly stated that analgesics were not used, and so it also follows that none justified a decision to withhold analgesics. We cannot distinguish with our methods those that used analgesia without mentioning it, from those that actively withheld analgesic medications (also without mentioning that). These 240 articles, along with the 62 that mentioned neither anesthesia nor analgesia, comprise 76% of our scored articles in which a reader could not know with any certainty whether analgesics were used or not.

### Date of Publication

To examine whether publication of 2010–11 guidelines for publishing animal data has had an impact on publication practices, we repeated the 6 mouse/rat models in April-May 2015, again using the order of addition to Pubmed as an approximation of recentness of publication. Date of publication had no effect on PC or AU for these models, suggesting that the 2011 ARRIVE guidelines are not yet having a significant impact on publication practices.

### Journals

We reviewed the 2013 impact factors and 2015 author guidelines for 184 journals. These journals published from 1 to 26 of the articles we reviewed (mean articles per journal = 2.3; median = 1). 2013 impact factors ranged from 0.234 to 33.116 (mean = 4.6). Four journals were too new in mid-2015 to have had impact factors calculated; these journals were excluded when we analyzed the correlation between impact factor and the articles’ PC and AU.

**Fig 7:**
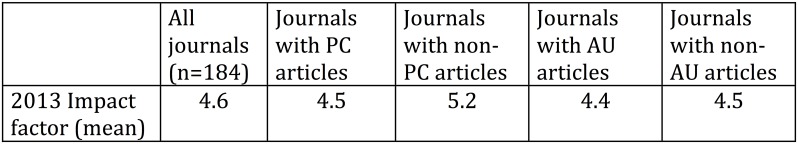
Impact Factors of Journals. Impact factors of journals with Publication-complete (PC), publication non-complete (non-PC), Analgesia Use (AU), and Analgesia non-Use (non-AU), in both the pre-2011 and the 2014–15 periods.

The 338 Publication Complete articles were in journals with an average impact factor of 4.5. (Fig 7) The 62 articles with a Publication Gap were in journals with an average impact factor of 5.2; impact factor did not differ by Publication Completeness (p > .05). 98 articles with Analgesic Use were in journals with an average impact factor of 4.4. 240 articles with an Analgesia Gap were in journals with an average impact factor of 4.5; impact factor did not differ by analgesia use or non-use (p > .05).

**Fig 8:**
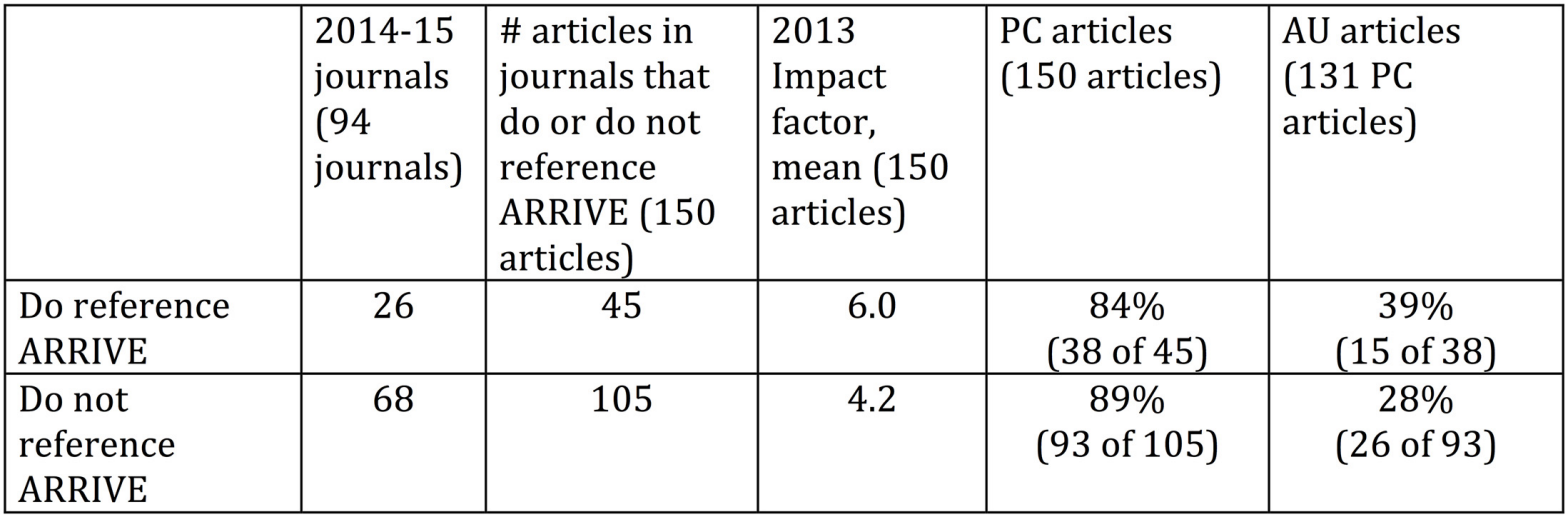
Comparison of Journals that Do or Do Not Reference ARRIVE guidelines. Mean 2013 impact factors of journals that do or do not reference the ARRIVE guidelines in their instructions for authors plus Publication Complete (PC) and Analgesia Use (AU) rates of 2014–15 articles in the journals.

Of the 94 journals publishing the 2014–15 articles, 26 journals, publishing 45 of the 150 articles, made direct or indirect reference to the 2010 ARRIVE guidelines. Of the 45 articles in journals that reference ARRIVE, 38 (84%) were Publication Complete, and of these 38, 15 (39%) were Analgesia Users. Of the 105 articles in journals that do not reference ARRIVE, 93 (89%) were Publication Complete, and of these 93, 26 (28%) were Analgesia Users. Impact factor did not differ by whether a journal referenced the guidelines (mean = 4.2 for no reference, mean = 6.0 for reference, p > .05). Reference to publication guidelines did not correlate with either PC or AU for the 2014–15 articles. (Fig 8)

### Adherence to CAMARADES checklist

The CAMARADES 10-point checklist for reporting neurological data in animals includes “avoidance of anaesthetics with marked intrinsic neuroprotective properties.” We scored 75 2014–15 articles in 3 neurological models (Fig 3) and found a range of anesthesia-analgesia combinations. CAMARADES does not list which anesthetics it considers neuroprotective, so it is not possible to score the 22 different descriptions of anesthetic-analgesic regimens against the CAMRADES checklist.

### Use of cited references

Of 400 scored articles, 163 required scoring 278 citations, comprising 174 separate papers one or more times. Seminal papers in some fields were cited multiple times.[37–39] In 98 cases, the referenced material provided no further anesthetic or analgesic information to change an article’s score. In 58 cases, an article with PC of 0 went to PC-without-AU (50 cases) or to PC-with-AU (8 cases). In 7 more cases, cited references provided analgesic information to an article that had specified anesthesia use but not analgesia. Without these cited references, overall PC would be 289 instead of 340, but AU among PC articles that mention anesthesia remains approximately 29%. In 25 papers, authors cited their own earlier first-author papers. In 12 of these 25, the cited papers added more anesthesia and/or analgesia information, and in one case the author specified a different anesthetic in scored and cited papers. In contrast to this, some authors, such as those who use the “spared nerve model” in rats (Models 10 and 16), cite earlier authors’ methods that include halothane, an anesthetic that is no longer commercially available; it is likely they used those authors’ surgical but not anesthetic methods.[38, 40]

### Qualitative Textual Observations

We found some publishing groups whose guidelines go beyond the ARRIVE and NAS guidelines. For example, the American Heart Association (AHA) instructions for authors of articles using animals include, “When describing surgical procedures, identify the preanesthetic and anesthetic agents used … For other invasive procedures on animals, report the analgesic or tranquilizing drug used. *If none were used*, *provide justification for exclusion*.” [emphasis added] Four AHA journals (*Arteriosclerosis*, *Thrombosis*, *and Vascular Biology; Circulation; Circulation Research;* and *Circulation*: *Heart Failure*) carried 10 of the 400 scored articles. Among these 10, PC was 90% and AU was 2/9 = 22%, which is slightly lower than the overall AU (~29%). Of the 7 articles with AU of 0, none clearly stated whether they excluded analgesic drugs or provided the required justification for exclusion.

In 2013, the Nature Publishing Group announced new efforts to improve reproducibility in their journals, with concern for when their authors “do not publish enough information for other researchers to assess results properly.” They abolished space restrictions in publication of the Methods sections, and developed their own checklist for authors and editors.[41] The *Nature* group does not mention animal anesthesia, analgesia or pain; they require a statement of ethics committee approval, and they “recommend consulting” the ARRIVE checklist.[42] Our 2014–15 dataset includes 5 articles published in 4 of Nature Publishing Group’s journals after announcement of the Publishing Group’s new standards (*Cancer Gene Therapy; Gene Therapy; Molecular Therapy; Scientific Reports*). One of these articles lists anesthesia and analgesia; the other four we scored with an “analgesia gap,” (AU = 0) with complete silence on whether analgesics were used or not.

Scientists vary in how explicitly they describe their animal experiments. Model #4 illustrates the range of ways in which scientists described their animal manipulations. This model (25 articles) includes placement of recording electrodes into the brains of nonhuman primates and surgically attaching “head posts” (rods affixed to the skull that will later serve to reduce head movements during behavioral or neurophysiological measurements in the unsedated animal). These major procedures require dissection through skin, muscle, periosteum, bone, and dura, typically with the animal’s head in a stereotactic device with metal bars that place pressure on the ear canals. They require surgical-plane anesthesia in one or more hours-long surgical sessions and an absolute minimum of brain injury, infection or inflammation. Their PC and AU of 60% and 40% were not significantly different from other models, but their description of surgery is worth noting. While one article describes in detail the tests they ran to rule out the effects of surgery, and presumably, anesthesia, on monkeys’ brain functions[43], 4 other articles describe collecting data from brain cells either with no mention of electrodes in the brain, or no mention that electrode placement is a complex surgery.[44–46]

While we did not score drug choices *per se*, we found several examples of drugs that we would expect to be challenged by a modern IACUC, such as chloral hydrate, diethyl ether, tribromoethanol, or sodium pentobarbital for surgical analgesia, and either subcutaneous lidocaine or systemic dipyrone/metamizole as sole agents for post-surgical analgesia.[47] We encountered some articles whose descriptions of anesthesia appeared either incomplete or inaccurate, such as those using agents no longer available (such as halothane) or those not stating pre-sedation for anesthesia of the larger species. It is almost impossible to establish inhalant or intravenous anesthesia in an adult rhesus monkey or a young adult pig without prior sedation with an intramuscular injection of a sedative drug, such as ketamine, tiletamine, azaperone or acepromazine. Despite this, 5 pig articles and 4 primate articles list their intravenous or inhalant anesthetics and no pre-anesthetic sedation.

## Discussion

In a range of biological fields, we found deficiencies in reporting on use of anesthesia for animal surgeries, and a significant lack of information on use of post-surgical analgesics. Pain and pain medications can affect the quality of data in animal experiments and also greatly affect animal welfare, and it was not possible in many cases to tell whether scientists withheld pain treatments or edited them out as methodologically irrelevant. Deficient reporting affects how well data can be evaluated or reproduced, but may also shape ongoing practice amongst scientists relying upon an incomplete body of literature.

In this project, we sampled what scientists read about animal pain management in their peers’ publications of experimental data. Whether as a methodological concern for reproducibility of published work, or as a guide for best practices in animal pain management, we found that the current literature in a range of fields is unreliable. Even in tracing back through 6 cited articles or more, 15% of scored articles did not yield any information on surgical anesthesia or post-surgical analgesia. Authors who cited using others’ methods did not generally specify that they used others’ pain management methods, as opposed to simply using their surgical techniques. Of those articles that did specify anesthesia, 71% provided no information on use of post-surgical analgesia. In total, 76% of articles were silent on whether animals who had undergone major survival surgery had received pain-relieving drugs or not.

We found comparable levels of uncertainty in all of the models we reviewed and in all the journals, regardless of their caliber. We did not find an effect of the 2010–11 guidelines on subsequent publication completeness in 6 rodent models, and articles in journals that use the ARRIVE guidelines were not more likely to mention their use of post-surgical analgesics. This is in line with Baker’s finding that the ARRIVE guidelines have not yet made an improvement in quality of reporting.[48] Overall, 76% of articles were silent on post-surgical analgesia, including no instances of authors explicitly stating that they did not provide pain medications for their animals.

When scientists confront a body of literature in which three fourths of articles are silent on pain management for major animal surgeries, what conclusions should they draw? Should they think pain was likely treated, but that pain and choice of analgesic drugs are methodologically irrelevant to data outcomes and need not be reported? Should they think that animal pain is solely an ethical concern, and that a statement of IACUC or ethics committee approval addresses the topic adequately? Or should they conclude that analgesic use is incompatible with some models, and that reviewers will reject manuscripts that include analgesics that they may consider unacceptable variables? Will they treat their own animals’ surgical pain or not?

Pain management is both a methodological concern and an animal welfare issue. We here explain how fuller reporting on pain management could improve the quality and reproducibility of science, and improve animal welfare.

### Pain management as a methodological concern in animal studies

The National Academies of Sciences guidelines call for full reporting of methods and substances administered., They note that anesthetics and analgesics can induce varied effects on studies, but are silent on the effects of untreated pain itself.[18] As the NAS, in another document, states: “Clearly, any drug *or* unintended physiologic state (e.g., pain, dehydration, acid base imbalance) that is introduced to an animal model *might affect* [emphasis added] the ultimate outcome.”[3] How strong an effect do pain-relieving drugs and untreated pain have on data? Is their effect consistent and strong enough that pain management should be in the top tier of methodological and reporting concerns, along with randomization, statistics and blinding?

Unalleviated surgical pain is a stressor and can affect immune function, food consumption, social behavior, metabolic state, all with potential impacts on data. Depending on the research questions being asked, judicious use of analgesics may actually counter the unwanted effects of untreated surgical pain, not just for animal well-being, but for data-quality too.[3]

Side-by-side comparisons of experiments conducted with different drug regimens are one way to define the direction and size of pain management’s effects, and such comparisons are available for some, but not all, models.[30, 31, 49–56] In some cases, outcomes are the same whether pain is treated or not.[56–58] In other cases, there is some effect, i.e., some difference in research outcome if pain is treated versus left untreated, though the magnitude of the effect needs to be examined. At this stage, such studies are scarce, and meta-analyses of the effects of pain and pain medications non-existent. Meta-analyses of published literature, however, can only determine how pain management might affect data if they have sufficient reporting of pain management details in the papers they are analyzing.

Scientists may assume, *a priori*, that when outcomes differ depending on whether analgesics were used or pain left untreated, that it must be the drugs, and not the untreated pain, affecting the data. This may often be wrong, or unknowable. As an example, several authors have looked at metastatic rates in rats following intravenous injection of tumor cells. They have found that the number of cells that lodge in the lungs and grow into cancerous nodules varies with whether the rats have undergone surgery, whether the surgical pain has been treated, and with which analgesic was used to treat the pain.[30, 31, 59, 60] Surgical pain depresses the immune system (allowing more metastasis) as do some analgesics; other analgesics combat this effect.[61, 62] Which is the “truer” model of human metastatic disease depends on the research question being asked. It is just as possible that the immunosuppression that surgical pain causes, not the various effects of pain medications, is skewing data.

While scientists in many fields should consider the effects of pain management decisions, neurobiologists have been particularly active in reviewing their field for publication biases that may affect data quality, reproducibility, and translatability.[14, 17, 21, 22, 26, 63–67] Given their focus on nerve and brain function, they should be the most attuned to the effects of neuroactive anesthetics and analgesics. Despite this, we found only the CAMARADES checklist to explicitly mention choice of anesthetics as a methodological concern. Its 10-point checklist for reviews of stroke models (which CAMARADES has now expanded to cover other neurological models as well) includes “avoidance of anaesthetics with marked intrinsic neuroprotective properties.”[21] Unfortunately, these guidelines mention ketamine alone as the neuroprotective anesthetic to avoid, but are silent about what other drugs they think might be problematic. They cite no systematic reviews to support their concern over the use of neuroprotective anesthetics; in fact, others before and since have found ketamine to be less neuroprotective than isoflurane or a fentanyl-fluanisone-midazolam combination.[65, 68] Moreover, CAMARADES, though one of the few guidelines to address research effects of surgical anesthetic choice, is silent on choice of post-surgical analgesics and of leaving pain untreated. These shortcomings in the CAMARADES guidelines, and in the literature on which they are based, are reflected in the wide range of anesthetic-analgesic combinations we found in 75 papers in 3 neurological models. (Fig 3)

The National Institute of Neurological Disorders and Stroke funded a project to replicate spinal cord injury findings in rodent laboratories, raising questions about just how perfectly any lab can replicate another’s methods of generating a spinal cord injury in rats or mice, either by reading their literature, or even through collaboration. They found “a surprising preponderance of failures to replicate,” and in their speculation of possible explanations, included anesthetics and post-injury analgesics as potential sources of uncontrolled variability. They note that individual IACUCs may not allow certain anesthetic-analgesic choices, even in the goal of replicating others’ works. If IACUCs do change anesthetic and analgesic practices, *and* these drugs (and unalleviated pain) have significant effects on outcomes in some experiments, then this would be a case in which welfare concerns could certainly shape research outcomes. But the IACUC and scientist are currently both working in the dark, when there are so few good data on how (and how much) pain and pain medications shape particular outcomes. Possibly, as the summary of the NINDS project questions: “A different anesthetic or drug could certainly alter the effect of a treatment, but if this occurs, can that treatment be considered robust?”[19] This is a testable question that is waiting to be tested. It is not testable if scientists do not report on their choices of anesthetics and analgesics.

As various professional groups work to develop review criteria and standardized procedures to improve reproducibility, better information on how pain management affects data is crucial. Jones *et al*. report on an interesting approach to improved reproducibility in cardiology research: establishing standardized protocols via multi-centric experiments using the same methods.[69] Their test case, defining cardiac infarct size with ischemic preconditioning in mice, rabbits and pigs, would allow other labs to use their methods and have a standardized lesion for comparison in their experiments. To their credit, they explicitly list the anesthetics and analgesics they use in each species. Their choices are questionable—such as barbiturate anesthesia for the surgeries in all three species, which veterinarians would not usually recommend—but we see the inclusion of anesthetic and analgesic details as an important step in standardizing these important variables across laboratories using these models. [70–72]

The large numbers of articles with no mention of anesthesia and/or analgesia, and the wide range of anesthetic-analgesic regimens in use in any given model, and the brief mentions of anesthesia/analgesia in publishing guidelines add up to suggest that neither scientists nor journal reviewers currently see anesthetic and analgesic choice as a significant methodological concern for quality of data.

Scientists’ low attention to publishing their pain management details stands in contrast to the number of projects in which scientists apply to their IACUC for approval to withhold pain medications. Annual reports to the USDA report some 8% of AWA-covered animals allowing unalleviated pain where pain-relieving drugs would adversely affect “the procedures, results, or interpretation of the teaching, research, experiments, surgery, or tests.” Though these figures do not include mice and rats, we see no reason to believe the percentages would be much different for them. Scientists, while in the planning phase of their experiments and while seeking IACUC approval, are highly cognizant of the methodological concerns in pain management choices, but in the writing phase those choices do not out-compete other methodological issues in word-limited publications.

### Pain management as a welfare concern in animal studies

As well as a methodological consideration, pain is also and most obviously a concern for animal welfare. It is a major focus of animal welfare regulations and of IACUC reviews.

The ARRIVE guidelines include as item #5 to indicate the “nature of the ethical review permissions […] that cover the research.”[16] Most journals’ instructions to authors directly or indirectly call on authors to state in their Methods what permissions they received. But as the NAS guidance document notes, “IACUC approval and animal facility accreditation are general indications of program quality but in no way obviate the need for proper description.”[18] We agree. Almost all of the articles we scored stated the animal-use approvals they had received, but still, 75% were silent on whether animals received pain medications after major surgery. There is no way for a reader to know whether the researchers, veterinarians and IACUC all agreed that pain-relieving drugs should be avoided, or that researcher, reviewers and editors agreed their use was unimportant. Scientists looking to evaluate or to replicate the published work, or the general public (whose taxes may have funded the project) both need more detail, not simply a statement of IACUC approval, to make their evaluations.

One important reason why a simple statement of IACUC or ethics committee approval is insufficient, at least in the United States, is the lack of clear guidance or standards in the rules that IACUCs follow. This is a particular concern when scientists believe they must request permission to deny post-operative pain-relieving drugs to animals. The USDA defines “Column E” (“pain involved; no drugs administered”) studies as those that cross a threshold of pain or distress that is “more than slight or momentary.” [73] A simple injection is not a “painful procedure,” but surgery is “considered a painful procedure in which pain is alleviated by anesthesia. Survival surgery *may* also require the use of peri-operative analgesia. [emphasis added]”[73] USDA’s Inspection Guide has guidance on how to categorize procedures on the annual report, but without guidance on how to report an animal who undergoes surgery with anesthesia and receives no post-surgical analgesics.[74] The *Guide for the Care and Use of Laboratory Animals* similarly fails to set a clear standard of practice for animal pain management, largely relying on veterinary consultation and IACUC review to assure the best animal welfare.[75, 76] Neither document states a clear standard of care that all animals undergoing major survival surgery should receive pain medications.

Neither the AWA nor the *Guide* sets a standard of what kinds of justification could permit withholding pain medications. In a recent survey for *The IACUC Handbook*, we (LC) found that IACUCs vary greatly in what they require of researchers who request approval to conduct painful surgeries without post-operative pain management. [77] Do IACUCs require a search for side-by-side comparisons of the same procedure with pain treated or not (the type of search we recommend as the gold standard)? Are they content to hear an investigator’s hypothetical concerns about how drugs might affect their data? USDA recommends “a database search as the most effective and efficient method for demonstrating compliance with the requirement to consider alternatives to painful/distressful procedures.”[4]

But such a search is no better than the literature base that it is searching. We are not confident that veterinarians or IACUCs have good tools for their role in the face of a body of literature that is so uninformative about pain management for particular research models, and a regulatory framework in which the standard of care is unclear.[75, 76] Certainly no veterinarian can offer evidence-based analgesic recommendations when the evidence in the literature is so sparse. Fuller reporting of anesthesia, analgesia and management of animals with unalleviated post-surgical pain, along with systematic reviews of pain management’s effects on data, could indeed make the AWA’s literature search not just efficient, but effective too.

Our concern, when 75% of the articles we reviewed have no mention either of treating pain or of leaving it untreated, is that scientists cannot know how to proceed in attempting to reproduce or adopt others’ methods and take silence about pain medications not just as evidence they are not being used, but that they cannot be used. Publishing pain management information may be a testimonial to the scientists’ attention to animal welfare, but it doubles its value when other scientists then read those details and incorporate those practices into their own studies.

### Are current guidelines adequate for the task?

In this review, we found animal pain to be largely invisible in published descriptions of animal experiments.

Few publishing guidelines or systematic reviews look at surgical anesthesia as a significant source of methodological bias, and even fewer consider analgesics or untreated pain. As one example of this, in their recent review of 77 2012 animal research articles in the field of critical care, Bara and Joffe split their work into two articles, one on methodological quality and the other on “the ethical dimension.” Anesthesia and pain management, which we argue should be in both papers, was relegated solely to the ethics paper.[9, 29] Indeed, pain management is not just a question of data integrity, but one of animal welfare too. But in the general silence around animal pain management as a methodological concern, it is relegated to “just” a welfare concern, the province of IACUCs and ethics committees, with journals’ and reviewers’ roles simply to tick the box verifying that the author lists the IACUC’s approval.

The ARRIVE and NAS guidelines, and many other systematic reviews of the quality of data of animal studies, emphasize the need for full and transparent reporting that allow for critical evaluation and reproduction of published data.[6, 15, 17, 64, 78, 79] Pain management has mostly stayed off their lists of criteria, overshadowed by randomization, concealment of allocation, blinding of researchers, and statistical analyses. ARRIVE tells scientists to “provide precise details of all procedures,” including “anaesthesia and analgesia used… rationale for specific choice of anaesthetic.” The ARRIVE checklist, however, lists 20 items, most of them composites of several sub-items, more than could conceivably fit into every research publication. Of most relevance to the present study, Item #7 (“Experimental Procedures”) calls for reporting formulation, route of administration and dose of all drugs (not just anesthetics and analgesics, though only analgesics include a requirement for monitoring), procedures (including surgery, behavioral tests), where the animals were tested, at what time of day, and what specialized equipment was involved.[16] Scientists cannot possibly fit full detail on all of ARRIVE’s checklist items into an article, or at least, not without a pages-long methods supplement that may be so detailed and hard-to-read that journals will reject it. They must edit, and currently, our review shows that pain management information is often edited out, or not considered for inclusion in the first place. Still, ARRIVE has been broadly marketed as *the* standard to which dozens of journals have signed on, and expanded attention to animal pain in subsequent updates of these guidelines could substantially improve pain management practices and reporting.

We believe that both those groups concerned with best publishing practices for quality reproducible data, and those promoting animal welfare, share much common ground. Both should promote full attention to and description of pain and pain medications’ potent roles in managing animal welfare and in influencing the data those animals produce. Pain is the “fifth vital sign” in 21^st^ century medical practice; in animal research, it is largely invisible.[80–83].

Best animal welfare goes hand-in-hand with best publication practices in reporting animal experiments, but how to improve on current practice? Eisen *et al*. say that journals, funders and authors are all accountable.[84] Baker *et al*. write that journals are not “enforcing” use of the ARRIVE guidelines.[48] Indeed, most journals that now endorse or use the ARRIVE guidelines and checklist have them more as a suggestion, than a requirement. The ARRIVE checklist currently includes 20 items, all of them composites of several smaller items. That’s already a large number of items, and not all of them are relevant to every animal experiment. A number of professional groups have started promoting discipline-specific publications guidelines, and like the NAS guidelines, many of them rely heavily on ARRIVE.[17, 69, 85–89] This is generally a good development, but we believe ARRIVE under-values the importance of pain management as a potential source of data bias, and is too quick to leave pain as a welfare concern in the hands of IACUCs, and that this shortcoming is being reproduced in discipline-specific guidelines. We recommend therefore that the next revision of the ARRIVE guidelines will explicitly call out pain management as a separate checklist item.

Laboratory animal welfare is a high enough societal concern to warrant two federal laws on animal care and use. We suggest that publication standards should be part of those federal regulations, not just recommendations for editors and journals. We view publication as part of a cyclical research process, not as some separate enterprise that happens after the research is done. The AWA and *Guide* treat animal experimentation as a linear activity that ends when the animals are dead. We see no good reason why the AWA and *Guide*’s standards for responsible laboratory animal use do not include standards for responsible reporting of that use. At the minimum, we would suggest that the Guide call on researchers who must withhold pain-relieving drugs to explicitly state and explain that in their published research. We see no reason why IACUCs would not read their scientists’ publications as part of their program for post-approval monitoring of animal use, to ensure both animal welfare and better data. Why is it left to journals to voluntarily develop and implement standards?

Animal pain, treated or not, still awaits systematic review of its effects on research outcomes, a review that will be challenged by the current poor reporting on pain management choices. With its methodological significance undefined, pain management is carved out from other potential sources of bias, as the province of IACUCs and ethics committees, and not the responsibility of peer reviewers during funding review or manuscript review.

## Conclusion

In this review of 400 published articles and ancillary material, we identified a serious deficiency in reporting on use of anesthetics and analgesics for laboratory animals undergoing major surgery in a range of experimental fields. Our review suggests that animal post-surgical pain is likely undertreated, but the literature is not sufficiently detailed to draw firm conclusions. Pain management still awaits systematic analysis of how much and in what direction pain and pain-relieving drugs might bias outcomes in various fields. Poor reporting of pain management challenges attempts to reproduce or evaluate published findings, or to perform the sorts of reviews that could identify just how much pain and pain-relieving drugs affect research outcomes. Under-reporting encourages under-treatment. It perpetuates itself when researcher scientists rightly or wrongly believe that analgesics are not used, and cannot be used, in their field, and then publish their own work with no mention of pain or pain medicines. The data suffer and the animals suffer. Whether journals, scientists, professional societies, IACUCs, regulators or others take the lead, clear standards for publication and clearer standards for animal protocol approvals are overdue.

## Supporting Information

S1 Data Spreadsheet of 400 Scored Articles. (XLSX)

S1 Fig Key to Data Spreadsheet. (DOCX)
