# Supplementary material for: Pain and Laboratory Animals: Publication Practices for Better Data Reproducibility and Better Animal Welfare
Source: PLoS One. 2016 May 12;11(5):e0155001. doi: 10.1371/journal.pone.0155001 (PMC4865140; doi:10.1371/journal.pone.0155001)
Supplement: S1 Fig — (DOCX) [file pone.0155001.s002.docx]

**Key to Data Spreadsheet**

**Shading**:

Unshaded: Publication Complete article + Analgesia Use (PC-AU score of 2)

Light Gray: Publication Complete with no mention of Analgesia Use (PC-AU score of 1)

Dark Gray: No mention of Anesthesia or Analgesia (Publication Not Complete) (PC-AU score of 0)

| **Column** | **Notes** |
| --- | --- |
| **Model and Article information** | |
| Model name | See Figure 1 of Text for description of the models. |
| Year | Date of Publication. Articles were grouped as either “pre-2011” or as “2014-15” for data analysis for effect of the 2010 ARRIVE guidelines. |
| Rodent or NonRodent | Pig, Primate and Dog are NonRodent.  Mouse, Rat, Hamster, Vole and Gerbil are Rodent. |
| Publication Complete (PC) | Score = 1 if authors mention surgical anesthesia and/or post-surgical analgesia (PC); 0 if they do not. |
| **Surgical Anesthesia Information** | |
| Anesthesia mentioned, but no specifics | Score = 1 if authors mention surgical anesthesia but do not name the anesthetic(s) used. Score is 0 if they used and named their anesthetics. N/A if they were not Publication Complete. |
| Anesthesia used and named | Score = 1 if authors mention surgical anesthesia and do name the anesthetic(s) used; 0 if they used anesthesia but do not name the anesthetics. N/A if they were not Publication Complete. |
| Names of anesthetics used | All anesthetic/analgesia drugs that were administered to the animal prior to the start of the surgery. N/A if anesthetics were not named. |
| **Post-operative Analgesia Information** | |
| Post-surgical Analgesia Used (AU) | Score is 1 if authors mention use of post-surgical analgesia. Score is 1 if long-acting analgesics are administered as part of surgical anesthesia. Score is 0 if article is Publication Complete but has no mention of post-surgical analgesia.  Score is N/A if article is Publication Not Complete. |
| PC-AU score | Score is 0 if article is not Publication Complete (PC); 1 if article is PC, but not an Analgesic User; 2 if PC and Analgesia Use (AU). Corresponds to dark gray, light gray and white shading of rows. |
| Analgesia but no specifics | Score is 1 if authors mention use of analgesia, but do not name the analgesic(s). Score is 0 if the analgesic(s) is/are named. N/A if article is Publication Not Complete or if article is Analgesic Non-Use. |
| Analgesia used and named | Score is 1 if the analgesic(s) is/are named. Score is 0 if authors mention use of analgesia, but do not name the analgesic(s). N/A if article is Publication Not Complete or if article is PC but with Analgesic Non-Use. |
| Names of Post-operative analgesic(s) used | Names, doses and duration of treatment of drugs used to provide post-surgical analgesia. Score is N/A if analgesics are not named. |
| Single analgesic class used | Score is 1 if analgesic(s) is/are named, and are in a single class of drugs. Score = 0 if there are 2 or 3 classes of analgesic drugs used. N/A if analgesics are not named. |
| Two analgesic classes used | Score is 1 if analgesics are named, and are in 2 classes of drugs. Score = 0 if there are 1 or 3 classes of analgesic drugs used. N/A if analgesics are not named. |
| Three analgesic classes used | Score is 1 if analgesics are named, and are in 3 classes of drugs. Score = 0 if there are 1 or 2 classes of analgesic drugs used. Score is N/A if analgesics are not named. |
| Multimodal analgesia score | Score is 1 if 2 or 3 analgesic classes are used and named. Score is 0 if a single analgesic class is named. Score is N/A if analgesics are not named. |
| Specify that analgesics were not used | Score is 1 if the article explicitly states that analgesics were not used. No articles received a score of 1 in this category. |
| Non-use of Analgesic explained | Score is 1 if authors stated that analgesics were not used, and explained why not. No articles explicitly stated that no analgesics were used, and so none explained a reason to withhold analgesia. Score is zero if article is PC but does not mention use of analgesics and N/A if article is Publication Non Complete. |
| Analgesia use during surgery (balanced anesthesia) | Score is 1 if drugs with analgesic activity were used during the surgery. See Figure 2 of text for names of analgesic drugs. Score is 0 if anesthetics are named and none of them are listed as having analgesic activity. N/A if anesthetics are not named. |
| Analgesia use during surgery, but no post-operative analgesia | Score is 1 if short-acting analgesic drugs were used during surgery, but the authors do not mention use of longer-acting or post-surgical drugs to provide post-surgical analgesia. See Figure 2 of text for names of analgesic drugs.  Score is 0 if no analgesic agents are part of surgical anesthesia OR if post-surgical analgesics were administered. N/A if anesthesia and/or analgesic details are not provided. |
| Extended analgesia used | Score is 1 if authors clearly state use of supplemental doses of analgesics after the initial peri-operative dose. Score is 0 if supplemental doses are not mentioned, including mention of “as needed” dosing with no evidence that animals did get further doses. N/A if analgesic details are not provided. |
| Analgesia Completeness (AC) | AC is calculated on a scale of 1-4 for articles that describe both their surgical anesthesia and post-surgical analgesia. Any mention of analgesia + Multimodal Analgesia + Analgesia use during surgery + Extended analgesia = AC. |
| **Journal Information** | |
| 2013 Impact Factor | Impact factors of journals; not available for four journals that were not in circulation long enough to have data to calculate an impact factor. |
| 2015 author instructions reference guidelines | Score is 1 if the journal’s posted instructions for authors state that authors should follow the ARRIVE publication guidelines and/or the National Academies of Sciences publication guidelines. Journals who refer authors to the EQUATOR guidelines, which reference the ARRIVE guidelines, received a score of 1. Score is 0 if the journal’s author instructions do not reference any of these publication guidelines. |
| **Use of Cited References** | |
| Needed cited references to score article | 400 primary articles were formally scored. Score is 1 in this column if an article that is Publication Non Complete or is PC but not an Analgesia Use article cites references that meet the original inclusion criteria (full text available in English). Score is 0 if the scored article includes complete anesthesia/analgesia information. |
| # of cited references required | If cited references were necessary to complete scoring an article, this column lists the numbers of cited references (including references they cite) to finalize the score on the primary article. |
| Change in score when cited articles are used | Use of cited references might: provide no additional information to change the initial score of the primary article (0); provide anesthesia-only information to change a Publication Non Complete article to PC with no analgesia use (1); provide anesthesia and analgesia information to change a Publication Non Complete article to PC with analgesia use (AU)(2); or to change a Publication Non Complete article with no analgesia use to a PC with AU article (3). |
